# Efficacy and Tolerability of Peritendinous Hyaluronic Acid in Patients with Supraspinatus Tendinopathy: a Multicenter, Randomized, Controlled Trial

**DOI:** 10.1186/s40798-017-0089-9

**Published:** 2017-06-05

**Authors:** César Flores, Ramón Balius, Guillermo Álvarez, Miguel A. Buil, Luisa Varela, Carlos Cano, Joaquín Casariego

**Affiliations:** 1 Department of Orthopaedic Surgery and Sports Medicine, Clínica CEMTRO, Madrid, Spain; 2 Department of Sports Medicine, Centre d’Estudis d’Alt Rendiment Esportiu (CEARE), Consell Català de l’Esport, Barcelona, Spain; 3 Department of Sports Medicine, AMS - Centro Médico del Ejercicio, Málaga, Spain; 4 Department of Sports Medicine, IVRE—Institut Valencià de Recuperació Esportiva, Valencia, Spain; 5 Department of Clinical Research, OPKO Health Europe, Plaza Europa, 13-15, Hospitalet de Llobregat, 08908 Barcelona, Spain; 6 Department of Physical Therapy, AMS—Centro Médico del Ejercicio, Málaga, Spain; 7 Medical Department, OPKO Health Europe, Barcelona, Spain

## Abstract

**Background:**

Physical therapy and peritendinous hyaluronic acid (HA) injections have both shown promising results in the treatment of shoulder tendinopathies. However, the superiority of treatment combining physical therapy and HA is unclear.

**Methods:**

Patients with ultrasound-confirmed supraspinatus tendinopathy were randomized to receive either physical therapy + subacromial HA injections or physical therapy only. Treatment efficacy was assessed using a Visual Analog Scale (VAS) for pain and an Activities of Daily Living (ADL) scale. Other measures were the number of rehabilitation sessions and days needed for recovery, the Tampa Scale for Kinesiophobia (TSK), and the physician and patient’s perception of efficacy and tolerability. Patients were followed up for 90 days.

**Results:**

Overall, VAS and ADL scores showed a progressive decrease during the follow-up (*P* < 0.01 at all visits for both groups), without significant differences between groups. The TSK score decreased significantly more in the HA group than in the control group (3.6 vs. 2.4; *P* < 0.001). Patients in the control group needed more rehabilitation sessions (28 vs. 22 in the HA group; *P* = 0.006) and more days for returning to their pre-injury activity (32 vs. 20 in the HA group; *P* = 0.013). Both patients and investigators perceived higher efficacy in the HA group than in the control group (*P* = 0.034). Both treatments were safe and well tolerated.

**Conclusions:**

Subacromial HA injections combined with physical therapy have high efficacy in the treatment of supraspinatus tendinopathy, leading to an earlier return to pre-injury activity and the need for fewer rehabilitation sessions, which may benefit both patients and the healthcare system.

**Electronic supplementary material:**

The online version of this article (doi:10.1186/s40798-017-0089-9) contains supplementary material, which is available to authorized users.

## Key Points

- Physical therapy is the gold-standard treatment for the management of tendinopathies.
- In this study, we found that combined treatment with 2% hyaluronic acid injections and physical therapy promotes faster recovery in patients with supraspinatus tendinopathy.
- Combined treatment with physical therapy and 2% hyaluronic acid may benefit the healthcare system and society by reducing rehabilitation costs and time off work.

## Background

Tendinopathy is a common injury in athletic populations, secondary to overuse [1, 2]. However, it may also occur in the overall population as a result of repetitive or excessive loading, and abnormal or unusual movements [3]. Also, metabolic- and hormone-related clinical conditions such as diabetes, menopause, and adiposity have been identified as systemic risk factors for tendinopathies [4–6]. Although the incidence of tendinopathies in the overall population has not been established, different authors have reported a rising trend associated with increasing interest in sport activities in high-income societies [1, 2, 7].

The first-line pharmacological treatment for tendinopathies is most often based on non-steroidal anti-inflammatory drugs (NSAIDs), which are useful for pain control but may cause patients to ignore warning symptoms, resulting in further damage to the affected tendon [3, 8]. Intratendinous injections of corticosteroids are also common in the standard management of tendinopathies and appear to successfully reduce inflammation and pain at short-term; however, the risk-benefit ratio of their use in the treatment of tendinopathies is currently controversial [7, 9]. Owing to the limitations of pharmacological treatments in modifying the structure of the tendon, the management of tendinopathies usually includes other non-pharmacological interventions with proven benefits in tendon recovery such as relative rest, cold, ultrasound, and physical therapy, specifically eccentric style exercises and stretching to prevent stiffness [1, 3]. Other non-pharmacological interventions proposed include shock wave therapy, iontophoresis, sclerotherapy, and nitric oxide patches; however, results are not very consistent and multicenter trials are needed to confirm their efficacy. Among all of the physical therapeutic interventions, eccentric strengthening has shown remarkably good therapeutic outcomes in the treatment of various tendinopathies such as those affecting the patellar, Achilles, supraspinatus, and wrist tendons [1, 10–14].

Among the recent advances in the management of tendon disorders, the peritendinous administration of hyaluronic acid (HA) has shown promising results in clinical trials including patients with tennis elbow [15], patellar tendinopathy [16], Achilles tendinopathy [17, 18], and various disorders involving tendons in the rotator cuff [19–22]. HA is the primary component of synovial fluid and provides the joint with lubrication and shock absorption [23]. Although the mechanisms of action in the treatment of tendinopathies are not well established, peritendinous injection of HA has shown to reduce tendon adhesion, provide mechanical protection, and upregulate the vascular endothelial growth factor and type IV collagen, resulting in the acceleration of tendon healing [24–26]. In the particular case of tendinopathies involving the rotator cuff, the use of HA injections led to a significant improvement of shoulder function in all published trials [19, 20, 22, 27, 28]. However, the superiority of HA over other interventions in scales assessing pain is controversial, and few studies have evaluated the efficacy of HA in the treatment of lesions affecting a particular tendon. In this regard, some authors have highlighted the need for further randomized trials to better establish which grade of lesions may benefit most from peritendinous injection of HA [27].

In this parallel-group, randomized, controlled trial, we investigated the efficacy and safety of peritendinous injection of HA in patients with persistent supraspinatus tendinopathy. To this end, we compared the therapeutic outcome of treatment with HA as an adjuvant to physical therapy with that of physical therapy as sole therapeutic intervention.

## Methods

### Study Design

This was a prospective, randomized, open-label, parallel-group, phase IV trial to explore the efficacy of subacromial injection of HA as adjuvant treatment for supraspinatus tendinosis. Patients were recruited between March 2014 and August 2015 from four Spanish centers, including one general hospital and three sports medicine centers. The study protocol was approved by the ethics committee of the Catalan Sports Council (Barcelona, Spain), and all patients gave their written informed consent before entering the study. All data were managed in agreement with local personal data protection law (LOPD 15/1999).

Upon entering the study, patients were randomized into two study groups using the Random Allocation Software (version 1.0): the *HA group* was treated with physical therapy + subacromial injection of HA (40 mg sodium hyaluronate/2 mL, OSTENIL^®^ TENDON, MW 1.6 MDa), and the *control group* was treated with physical therapy only. The exercise plan within the physical therapy standard of care (provided in the Additional file 1) was the same for both groups, and it was performed at a physiotherapy center under individual guidance. All patients started with three sessions per week, and the schedule was then tailored at physician discretion on the basis of patient progress. The physical therapy did not include any intervention based on physical energy. Patients in the HA group received two ultrasound-guided, subacromial bursa injections at the baseline visit and at day 7. Injections were performed with the patient in a sitting position and the injured arm on the back, in internal rotation. After identifying the inflamed subacromial bursa in the ultrasound image, the needle was inserted between the two bursa layers and the HA was gently injected. In the case of presence of liquid inside the bursa, a small amount of liquid was drawn before injecting the HA. All participants were urged not to undertake physical labor (e.g., bearing weight, practicing sports, overstraining, etc.) with the affected arm during the follow-up period. Concomitant treatment with NSAIDs (400 mg ibuprofen up to 1200 mg daily, according to the routine practice of participant centers) was permitted. Patients were followed up at days 7, 15, 30, and 90 after starting treatment. The patient discharge was considered at physician discretion based on the results of the ultrasound scanning.

### Study Population

The study population comprised subjects aged between 18 and 60 years with chronic, ultrasound-confirmed supraspinatus tendinopathy of at least 4 weeks duration and referring moderate pain (VAS score ≥6). Recruitment was limited to active subjects (i.e., those who performed daily physical labor in their workday and/or attended the gym at least 2–3 times a week, where they performed an aerobic activity for at least 50 min during each session, with at least three sessions per week). Patients who had undertaken physical rehabilitation for the same injury in the 6 months prior to the study were excluded from the record. Other exclusion criteria were the presence of systemic diseases, previous shoulder surgery, radiology-confirmed acromion type III in the Bigliani scale [29], and other shoulder injuries: fracture or dislocation, adhesive capsulitis, calcifying tendinopathy, total or partial rotator cuff tears, and acromioclavicular and/or glenohumeral joint diseases.

### Study Endpoints and Variables

The primary endpoint was the assessment of efficacy using the American Shoulder and Elbow Surgeons (ASES) standardized shoulder assessment form [30–32]. The ASES form includes the evaluation of pain by means of the Visual Analog Scale (VAS) and the Activities of Daily Living (ADL) scale, which scores the difficulties for performing 10 everyday activities between 0 and 3. Secondary endpoints included indirect measures of efficacy such as the Tampa Scale for Kinesiophobia (TSK) [33], the number of rehabilitation sessions needed for recovery (i.e., absolute discharge, confirmed by ultrasound imaging, and return to daily activities, work and/or sporting activities), the number of days required to return to work or to sport activity, and physician and patient perception of the efficacy of the treatment received (rated on a 0–4 scale, where 0 was “very poor” and 4 was “excellent”). In addition to recording all adverse events occurring during the follow-up, the perception of patients and physicians on treatment tolerability was assessed on a 4-point scale. All variables were assessed at each visit.

### Statistical Analysis

The sample size was estimated considering a precision in the mean ASES score of 14.5 (average between 12 and 17, considered the minimum difference in the mean score to detect a relevant change in patients with rotator cuff disease [34]), and a standard deviation (SD) of 17.5 (the average reported in previous studies [30, 34]). Considering these assumptions, a study sample of at least 31 patients per group would provide a statistical power of 90% with a significance α level of 0.05.

The analysis was planned as an intention to treat analysis; hence, patients lost to follow-up after the first visit were included in the analysis. Continuous variables were summarized using means and measures of variance, whereas categorical variables were summarized using frequencies and percentages. The means of the baseline characteristics in each group were compared using the *T* test, whereas the percentages were compared using Fisher’s exact test. The differences in scores of the efficacy scales between the baseline and the other study visits within each group were assessed using the *T* test for paired samples, whereas differences between the study groups throughout the study were compared using an ANCOVA test. On the other hand, the perceptions of physicians and patients of tolerability and efficacy in each study group were compared using the non-parametric Mann-Whitney *U* test. The analysis was performed on available data only, and thus no imputations were done for missing values, which were eliminated pairwise. The significance threshold was set at a α value of 0.05, and all analyses were computed by an external statistical team (Onmedic) using the SAS System statistics software (Version 9.4.).

## Results

### Patient Characteristics

**Fig. 1:**
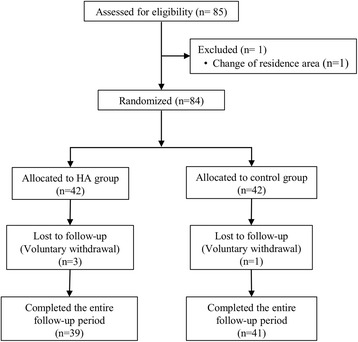
Flow diagram of the patients included in the study

Of 84 patients starting the study, 42 were allocated to the treatment group and 42 to the control group (Fig. 1). All patients assigned to the HA group successfully received treatment with HA according to the established schedule. Patients who were lost to follow-up withdrew from the study after visit 3 (one patient) and visit 5 (three patients) referring no specific reasons for study withdrawal.

At baseline, patients allocated to the HA group and the control group had no significant differences in their demographic characteristics (Table 1). Regarding clinical characteristics, the scores of all scales used in the efficacy assessment were equal between the two groups, except the TSK, which was significantly higher in the HA group (25.4 [4.1] and 29.8 [4.3] mean [SD] for the control and the HA group, respectively; *P* < 0.001). In this scale, questions relating to the perceived threat of getting a poorer health condition displayed the greatest differences between groups. The overall baseline scores in the VAS and ADL scales indicated a moderate affectation grade in our study patients. Most patients had taken NSAIDs during the month prior to the study start, but in most cases, consumption was occasional.

**Table 1:** Baseline characteristics of study patients (percentages of patients in categorical variables were calculated for each group)

	Overall	HA group	Control group	*P* value^a^
Demographic characteristics				
Age (years), *mean (SD)*	40.2 (12.4)	42.3 (11.5)	38.0 (13.1)	0.113
BMI (kg/m^2^), *mean (SD)*	24.5 (3.3)	24.6 (3.31)	24.5 (3.3)	0.886
Sex, *n (%)*				
Males	44 (53%)	24 (59%)	20 (48%)	0.382
Females	39 (47%)	17 (41%)	22 (52%)	
Clinical characteristics				
Affected shoulder, *n (%)*				
Right	52 (62%)	27 (64%)	25 (60%)	0.822
Left	32 (38%)	15 (36%)	17 (40%)	
VAS, *mean (SD)*	7.22 (0.95)	7.33 (0.88)	7.11 (1.01)	0.286
ADL, *mean (SD)*	17.2 (4.3)	16.9 (3.7)	17.5 (4.7)	0.491
TSK, *mean (SD)*	27.6 (4.7)	29.8 (4.3)	25.4 (4.1)	<0.001
NSAID consumption during the last month, *n (%)*				
Any consumption	43 (51%)	24 (57%)	19 (45%)	0.382
Frequency				
Often	8 (10%)	6 (14%)	2 (5%)	0.281
Occasionally	35 (42%)	18 (43%)	17 (40%)	

^a^The *P* values correspond to the comparison between the HA group and the control group (*T* test for quantitative variables and Fisher’s test for categorical variables)

### Main Efficacy Scales

**Fig. 2:**
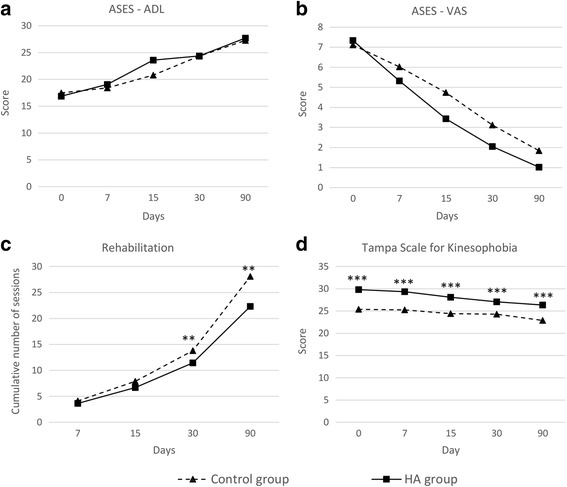
Main efficacy outcomes. *ASES* American Shoulder and Elbow Surgeons standardized shoulder assessment form: *ADL* Activities of Daily Living domain (**a**), *VAS* Visual Analogue Scale domain (**b**). Number of rehabilitation sessions needed for recovery (**c**). Tampa Scale for Kinesophobia (**d**). **p < 0.01 for control vs. HA groups comparison. ***p < 0.001 for control vs. HA groups comparison

Patients in both groups experienced a significant improvement in the VAS and ADL scores during the follow-up period (*P* < 0.01 paired *t* test in all visits for both scores in both intervention groups). After 90 days of follow-up, the mean VAS score decreased 5.3 (SD 2.1; 95% CI 4.6–6.0) points in the control group and 6.4 (SD 1.54; 95% CI 5.9–6.9) points in the HA group; the corresponding decline in the ADL score was 9.8 (SD 6.0; 95% CI 7.9–11.6) and 11.0 (SD 4.7; 95% CI 9.5 – 12.5) points in the control and the HA group, respectively. However, the comparative analysis of the VAS and the ADL score between groups did not reveal significant differences between patients treated with HA and physical therapy and those treated with physical therapy only (Fig. 2a, b).

### Indirect Measures of Efficacy

**Fig. 3:**
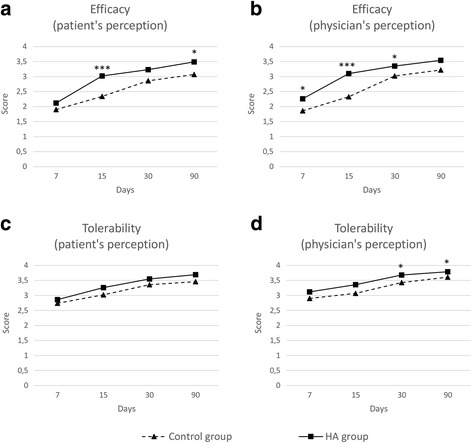
Perceived efficacy (**a**, **b**) and tolerability (**c**, **d**) outcomes, assessed on a 4-point Likert scale. *p < 0.05 for control vs. HA groups comparison. **p < 0.01 for control vs. HA groups comparison. ***p < 0.001 for control vs. HA groups comparison

In addition to the assessment of scales related to function and pain, we investigated the impact of treatment on indirect measures of efficacy such as the perceived threat of movement and pain (by means of the TSK scale), number of rehabilitation sessions and number of days needed for complete recovery, and patient and physician perception of efficacy. TSK scores were significantly higher in the HA than in the control group in all visits (Fig. 2d). However, patients in the HA group experienced a significant improvement compared to baseline from day 15 (*P* < 0.001 for comparisons between baseline and visits at days 15, 30, and 90), whereas in the control group, the variation of the TSK score from baseline was only significant at day 90. In addition, the decrease in TSK scores from baseline was significantly greater in the HA group than in the control group from day 7 (*P* < 0.001 in all ANOVA paired test for comparisons at visits at days 7, 15, 30, and 90). After 90 days of follow-up, the decline in mean (SD) TSK score was significantly greater in the HA group: the mean TSK score decreased 2.4 (SD 4.2; 95% CI 1.1–3.8) in the control group and 3.6 (5.1; 95% CI 1.9–5.2) in the HA group from baseline to day 90 (*P* < 0.001). The cumulative number of rehabilitation sessions was significantly greater in the control group at visits at days 30 and 90 (Fig. 2c). At the end of the follow-up period, patients in the control group needed a mean of 28 (SD 8; 95% CI 26–31) sessions, whereas those in the HA group accounted a mean of 22 (SD 10; 95% CI 19–26) sessions (*P* = 0.006). On average, patients in the HA group returned 12 days earlier to their pre-injury activity (either work or sport) than those in the control group (mean days was 32 [SD 21; 95% CI 26–39] in the control group and 20 [SD 21; 95% CI 13–27] in the HA group; *P* = 0.013). Treatment efficacy was also assessed by means of patient and physician perception of each treatment. Patients in the HA group rated the efficacy as higher than those in the control group in all visits; however, the differences in mean scores between groups were only significant at days 15 and 90 (Fig. 3a). At the end of the follow-up period, the mean scores (on a 4-point Likert scale) were 3.1 (SD 1.0; 95% CI 2.8–3.4) and 3.5 (SD 0.8; 95% CI 3.2–3.7) in the control and the HA group, respectively (*P* = 0.034). Accordingly, investigators considered treatment with HA plus rehabilitation to be more efficacious during the entire follow-up; in this case, significant differences with the treatment based solely on physical therapy were found at days 7, 15, and 30 (Fig. 3b). Lastly, the NSAID consumption showed a progressive trend towards a reduction during follow-up in the overall group (from 39 patients taking NSAIDs at baseline to 16 patients at day 90). The percentage of patients who reported any NSAID consumption at visits 2 to 5 were 50, 31, 15, and 18% in the HA group and 43, 33, 21, and 22% in the control group. However, no significant differences were observed between groups at any visit.

### Tolerability and Safety

Based on patient perception, treatment with HA injections and physical therapy was tolerated equally to that based on physical therapy only (Fig. 3c). Accordingly, investigators rated the tolerability of both interventions equally during the first 15 days of treatment but considered that at days 30 and 90, treatment with HA and physical therapy had greater tolerability than physical therapy only (Fig. 3d).

During the follow-up period, 10 patients (12%) reported 13 adverse events, all of which were considered mild: 5 in the HA group, and 8 in the control group. Of 5 adverse events reported in the HA group, 4 were found to be related to treatment with HA, all of them pain after injection.

## Discussion

In this multicenter, parallel-group, randomized trial including patients with supraspinatus tendinopathy, we found that both physical therapy alone and physical therapy combined with subacromial HA injections had good tolerability and resulted in significant pain reduction and successful functional recovery. When compared to physical therapy only, patients treated with physical therapy and HA returned significantly earlier to work and needed fewer rehabilitation sessions.

The study groups were well balanced regarding the baseline demographic and clinic characteristics, including the prevalence of the affected shoulder, which was mostly the right one, as expected for an overuse injury. NSAID consumption was greater in the HA group; however, no significant differences arose between the two groups. Likewise, we did not observe significant differences in the baseline scores of the assessed scales, except the TSK, which was significantly higher in the HA group. The TSK has been little used in the assessment of HA treatment efficacy and measures the perceived threat of movement and expected pain in injured patients. In this regard, the fear of an injected treatment (not existing in the control group, as no placebo treatment was administered) could explain the discrepancy between the different TSK score and the homogeneity in other baseline measures of pain, such as the VAS score or NSAID consumption. Overall, the baseline scores of the TSK, VAS, and ADL scales revealed a moderate severity of the tendinopathy in our study patients.

After 90 days of follow-up, patients in both groups experienced a significant improvement in the VAS, ADL, and TSK scores, irrespective of the intervention received. This result is consistent with previous studies proving that treatment based solely on eccentric strengthening is sufficient for mid-term improvement in pain and function [1]. When compared with physical therapy only, we found that the addition of treatment with subacromial HA significantly reduced the number of rehabilitation sessions and the treatment days needed for recovery. The reduction in the TSK score during the follow-up period was also greater and occurred earlier in the HA group, although the baseline differences between scores in each group might have influenced the differences observed during follow-up. In line with the better therapeutic outcome observed in the HA group, both patients and physicians rated significantly higher the efficacy of the combined treatment of physical therapy and subacromial HA than physical therapy only. In the case of physician perception, combined treatment was rated significantly higher than physical therapy from the first visit (day 7).

In light of the differences observed between study groups in the indirect measures of efficacy, we would also expect more remarkable differences between groups in treatment outcomes related to pain and the ability to perform ADL. However, NSAID consumption and the assessment of the VAS and ADL scores did not reveal significant differences between patients treated with physical therapy only and those treated with physical therapy and subacromial HA injections. Of note, as a clinical study performed on a medical device, the doses of concomitant medicines were not recorded, and therefore, no dose adjustment was performed in the comparative analysis. Considering the proven efficacy of physical therapy in the treatment of tendinopathies of the rotator cuff, it seems reasonable that a larger sample size would provide clearer differences between groups. Nevertheless, the superiority of HA treatment in pain improvement is unclear, and results in previous studies assessing the efficacy of HA injections in patients with tendinopathies affecting the rotator cuff are inconsistent. Merolla et al. found that HA monotherapy significantly reduced pain in more visits than physical therapy only [19], whereas Sengul et al. reported similar pain scores in patients receiving both interventions [22]. Similar discrepancies have been found when investigating the efficacy of a combined treatment using HA injections and physical therapy. Thus, Meloni et al. concluded that rehabilitation exercises and HA injections were significantly more efficacious in pain reduction than the same exercises accompanied with placebo injections containing sodium chloride [20]. On the other hand, Ozgen et al. compared the efficacy of HA injections with that of physical therapy and found no differences in pain improvement in most of the follow-up visits [28]. Of note, the baseline VAS scores reported by Ozgen et al. in the study sample were remarkably small (below 1), which may have contributed to narrow the differences between groups. On the other hand, the ADL score has been barely assessed in trials investigating the efficacy of injected HA in patients with tendinopathies of the rotator cuff. In our study, all participants received clear instructions not to undertake physical labor with the affected arm until recovery. Such indication, not included in pivotal trials assessing the reliability of the ASES score [30, 31], allowed us to limit the bias related to the different jobs and activities performed by each patient; however, it was likely to influence the overall ADL score, as patients may have limited their day-to-day activities, regardless of their functional recovery and pain.

In line with safety data previously reported in the literature [19, 20, 22], both interventions were safe and well tolerated, with a 12% rate of adverse events rate—all of them mild—, mostly reported by patients in the control group. Furthermore, both patients and physicians rated tolerability over 2.5 points on a 4-point Likert scale during the entire follow-up. Perceived tolerability progressively increased throughout the follow-up period, and no significant differences were observed between the study groups.

The main limitation of our study is the lack of control groups. Unlike other studies, aimed at investigating the efficacy of HA injections alone, our objective was to compare the combined treatment with the gold-standard for the management of tendinopathies. The presence of a placebo group with non-active peritendinous injections might have limited possible biases, particularly in patient-reported efficacy. Accordingly, the inclusion of a sham physical therapy would have yielded more extreme differences between groups. However, including more than two intervention groups would have dramatically reduced the size of each group. In fact, the final sample size (84 patients) was larger than that in most studies assessing the efficacy of HA injections in tendinopathies of the rotator cuff [27], but it still limited the observation of significant differences in some scales. Along the same lines, the open-label HA administration may introduce a bias in both the physician and patient efficacy assessment. Finally, due to the sample size limitations, all centers were considered as a whole and no cluster analyses were performed to explore the center bias.

## Conclusions

Despite the limitations of our study, the results obtained in most of the assessed scales are consistent and support the use of subacromial HA injections as adjuvant treatment to physical therapy in the management of supraspinatus tendinopathy. In our experience, treatment based only on physical therapy was sufficient to reduce pain and improve function. In addition, the combination of physical therapy with subacromial HA reduced the number of rehabilitation sessions and recovery time. These findings suggest that combined treatment with physical therapy and HA may not only increase patients’ quality of life but it might also benefit the healthcare system and society by reducing rehabilitation costs and time off work.

## Additional file

Additional file 1: Rehabilitation protocol. (PDF 681 kb)

## References

[1] Andres BM, Murrell GAC (2008). Treatment of tendinopathy: what works, what does not, and what is on the horizon. Clin Orthop Relat Res.

[2] Renstrom P, Woo SL (2008). Tendinopathy: a major medical problem in sport. Tendinopathy in athletes.

[3] Kaux JF, Forthomme B, le Goff C, Crielaard JM, Croisier JL (2011). Current opinions on tendinopathy. J Sport Sci Med.

[4] Frizziero A, Vittadini F, Gasparre G, Masiero S (2014). Impact of oestrogen deficiency and aging on tendon: concise review. Muscles Ligaments Tendons J.

[5] Oliva F, Piccirilli E, Berardi AC, Frizziero A, Tarantino U, Maffulli N (2016). Hormones and tendinopathies: the current evidence. Br Med Bull.

[6] Frizziero A, Salamanna F, Della Bella E, Vittadini F, Gasparre G, Aldini NN, et al. The role of detraining in tendon mechanobiology. Front Aging Neurosci. 2016;8:43. doi:10.3389/fnagi.2016.00043. 10.3389/fnagi.2016.00043 PMC4770795 26973517

[7] Maffulli N, Longo UG, Loppini M, Spiezia F, Denaro V (2010). New options in the management of tendinopathy. Open access J Sport Med.

[8] Magra M, Maffulli N (2006). Nonsteroidal antiinflammatory drugs in tendinopathy: friend or foe. Clin J Sport Med.

[9] Coombes BK, Bisset L, Vicenzino B (2010). Efficacy and safety of corticosteroid injections and other injections for management of tendinopathy: a systematic review of randomised controlled trials. Lancet.

[10] Reinking M (2012). Tendinopathy in athletes. Phys Ther Sport.

[11] Stanish WD, Rubinovich RM, Curwin S (1986). Eccentric exercise in chronic tendinitis. Clin Orthop Relat Res.

[12] Jonsson P, Alfredson H (2005). Superior results with eccentric compared to concentric quadriceps training in patients with jumper’s knee: a prospective randomised study. Br J Sports Med.

[13] Bahr R, Fossan B, Loken S, Engebretsen L (2006). Surgical treatment compared with eccentric training for patellar tendinopathy (Jumper ’ s Knee). J bone Jt Surg.

[14] Dimitrios S, Pantelis M, Kalliopi S (2012). Comparing the effects of eccentric training with eccentric training and static stretching exercises in the treatment of patellar tendinopathy. A controlled clinical trial. Clin Rehabil.

[15] Petrella RJ, Cogliano A, Decaria J, Mohamed N, Lee R (2010). Management of tennis elbow with sodium hyaluronate periarticular injections. Sports Med Arthrosc Rehabil Ther Technol.

[16] Muneta T, Koga H, Ju YJ, Mochizuki T, Sekiya I (2012). Hyaluronan injection therapy for athletic patients with patellar tendinopathy. J Orthop Sci.

[17] Kumai T, Muneta T, Tsuchiya A, Shiraishi M, Ishizaki Y, Sugimoto K (2014). The short-term effect after a single injection of high-molecular-weight hyaluronic acid in patients with enthesopathies (lateral epicondylitis, patellar tendinopathy, insertional Achilles tendinopathy, and plantar fasciitis): a preliminary study. J Orthop Sci.

[18] Petrella MJ, Cogliano A, Petrella RJ (2009). Original research: long-term efficacy and safety of periarticular hyaluronic acid in acute ankle sprain. Phys Sportsmed.

[19] Merolla G, Bianchi P, Porcellini G (2013). Ultrasound-guided subacromial injections of sodium hyaluronate for the management of rotator cuff tendinopathy: a prospective comparative study with rehabilitation therapy. Musculoskelet Surg.

[20] Meloni F, Milia F, Cavazzuti M, Doria C, Lisai P, Profili S (2008). Clinical evaluation of sodium hyaluronate in the treatment of patients with sopraspinatus tendinosis under echographic guide: experimental study of periarticular injections. Eur J Radiol.

[21] Kim YS, Park JY, Lee CS, Lee SJ (2012). Does hyaluronate injection work in shoulder disease in early stage? A multicenter, randomized, single blind and open comparative clinical study. J Shoulder Elb Surg.

[22] Sengul I, Oz B, Yoleri O, Olmez N, Memis A, Uluç E. Sodium hyaluronate injections compared to local modalities for the treatment of shoulder impingement syndrome/Omuz sikisma sendromu tedavisinde sodyum hiyaluronat enjeksiyonu ile lokal modalitelerin karsilastirilmasi. Turk J Phys Med Rehab 2008; 54:138–43.

[23] Moreland LW (2003). Intra-articular hyaluronan (hyaluronic acid) and hylans for the treatment of osteoarthritis: mechanisms of action. Arthritis Res Ther.

[24] Riccio M, Battiston B, Pajardi G, Corradi M, Passaretti U, Atzei A (2010). Efficiency of Hyaloglide in the prevention of the recurrence of adhesions after tenolysis of flexor tendons in zone II: a randomized, controlled, multicentre clinical trial. J Hand Surg Eur Vol.

[25] Yagishita K, Sekiya I, Sakaguchi Y, Shinomiya K, Muneta T (2005). The effect of hyaluronan on tendon healing in rabbits. Arthrosc J Arthrosc Relat Surg.

[26] Halici M, Karaoglu S, Canoz O, Kabak S, Baktir A, Petrella MJ (2004). Sodium hyaluronate regulating angiogenesis during Achilles tendon healing. Knee Surg Sport Traumatol Arthrosc.

[27] Osti L (2015). Clinical evidence in the treatment of rotator cuff tears with hyaluronic acid. Muscles Ligaments Tendons J.

[28] Ozgen M, Fırat S, Sarsan A, Topuz O, Ardıç F, Baydemir C (2012). Short- and long-term results of clinical effectiveness of sodium hyaluronate injection in supraspinatus tendinitis. Rheumatol Int.

[29] Bigliani LU (1986). The morphology of the acromion and its relationship to rotator cuff tears. Orthop Trans.

[30] Kocher MS, Horan MP, Briggs KK, Richardson TR, O’Holleran J, Hawkins RJ (2005). Reliability, validity, and responsiveness of the American Shoulder and Elbow Surgeons subjective shoulder scale in patients with shoulder instability, rotator cuff disease, and glenohumeral arthritis. J Bone Jt Surg Am.

[31] Richards RR, An KN, Bigliani LU, Friedman RJ, Gartsman GM, Gristina AG (1994). A standardized method for the assessment of shoulder function. J Shoulder Elb Surg.

[32] Roy JS, Macdermid JC, Woodhouse LJ (2009). Measuring shoulder function: a systematic review of four questionnaires. Arthritis Care Res.

[33] Gómez-Pérez L, López-Martínez AE, Ruiz-Párraga GT (2011). Psychometric properties of the spanish version of the Tampa Scale for kinesiophobia (TSK). J Pain.

[34] Tashjian RZ, Deloach J, Green A, Porucznik CA, Powell AP (2010). Minimal clinically important differences in ASES and simple shoulder test scores after nonoperative treatment of rotator cuff disease. J Bone Jt Surg.

