# Supplementary material for: Efficacy and Tolerability of Peritendinous Hyaluronic Acid in Patients with Supraspinatus Tendinopathy: a Multicenter, Randomized, Controlled Trial
Source: Sports Med Open. 2017 Jun 5;3:22. doi: 10.1186/s40798-017-0089-9 (PMC5459785; doi:10.1186/s40798-017-0089-9)

Rehabilitation protocol - exercises

The rehabilitation protocol is structured in two sections, corresponding to (1) exercises aimed at improving flexibility and restoring the range of motion of the rotator cuff, and (2) exercises aimed at strengthening muscles of the scapula and the rotator cuff.

Patients should perform all exercises in every session. The rehabilitation program starts with three sessions weekly, which may then increase at physician discretion.

Flexibility and range of motion

Except for the first exercise, two sets of each exercise should be performed. In each set, the patient will hold the stretch for 30 seconds and then relax for 10 seconds.

1. Pendulum exercise. Let the injured arm hanging as relaxed as possible, and then move it in a circular motion: 20 times in clockwise circles and 20 times in counterclockwise circles.

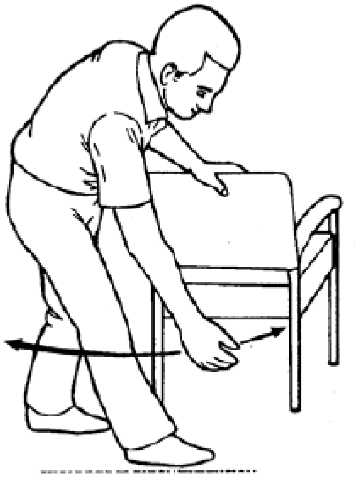

2. Scapular retraction. Lift the shoulders and pull them backward. Hold this posture while pinching the medial border of the scapulae together.

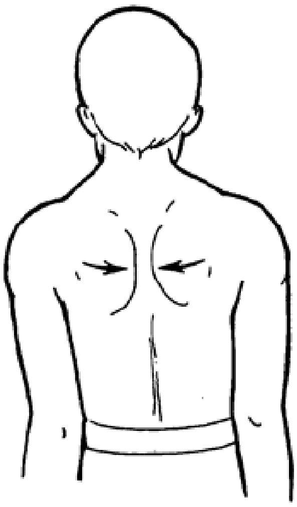

3. Posterior capsule stretch. Bring the affected arm towards the healthy shoulder while flexing the elbow 90º. Use the hand or forearm of the healthy arm to pull the injured one towards the thorax.

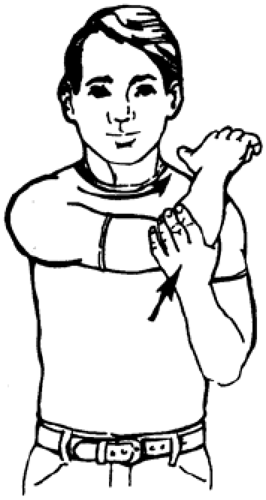

4. Trapezius stretch. In a sitting position, place the injured arm on the back in internal rotation and use the healthy arm to gently move the head towards the healthy shoulder.

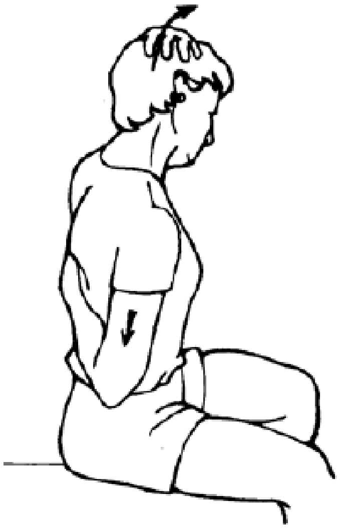

Strengthening

Two sets of 10 repetitions will be performed in each exercise.

5. Internal and external rotation (0º flexion). Tie the *theraband* at elbow level. Internal and external rotations (two different exercises) will be performed with the elbow in 90º flexion and the wrist straight.

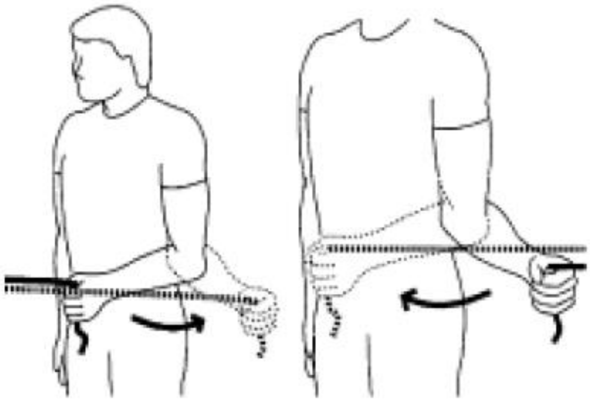

6. Row. Tie the *theraband* at elbow level and keep elbows in 90º flexion. Pull the elbows straight back while pinching the medial border of the scapulae together.

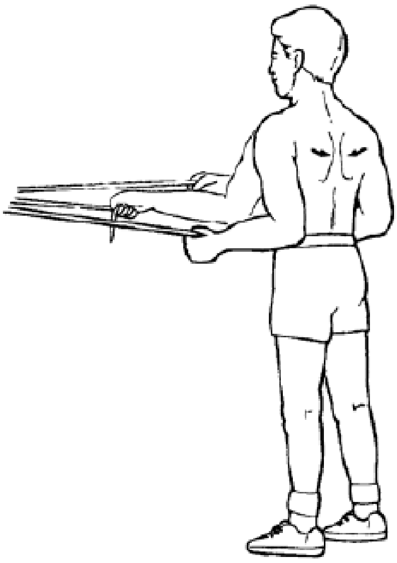

7. Lower trapezius. Tie the *theraband* at extended elbow level. While keeping elbows extended, pull the arm straight back while pinching the medial border of the scapulae together.

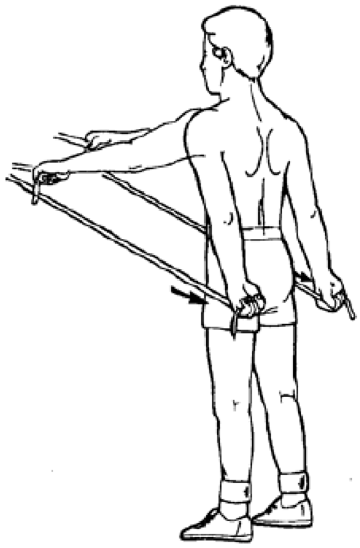

Supplement: Additional file 1: — Rehabilitation protocol. (PDF 681 kb) [file 40798_2017_89_MOESM1_ESM.pdf]
